# Perceptions of Cardiovascular Healthcare Professionals Regarding Clinical Trials: A Survey-Based Study from the Middle East

**DOI:** 10.5334/gh.1389

**Published:** 2025-01-17

**Authors:** Zainab Atiyah Dakhil, Hasan Ali Farhan, Mohammed Dheyaa Marsool, Mohammed Saad Qasim, Michele Peters, Jose Leal

**Affiliations:** 1 Ibn Al-Bitar Cardiac Centre, University of Baghdad/Al-Kindy College of Medicine, Iraq; 2 Iraqi Scientific Council of Cardioloy, University of Baghdad/College of Medicine, Iraq; 3 University of Baghdad/Al-Kindy College of Medicine, Iraq; 4 University of Oxford, UK

**Keywords:** Developing countries, trial, diversity, inclusion, evidence-based, Iraq

## Abstract

**Background:**

Low-middle income countries harbor the highest burden of cardiovascular diseases globally, but there is an under-representation of these countries in cardiovascular clinical trials. This limits the generalizability of the trial results to these countries. There is a lack of data on insights of cardiologists in these countries regarding conducting and participating in clinical trials. We sought the views of cardiovascular healthcare professionals in Iraq on participation in clinical trials.

**Method:**

Cardiovascular professionals in Iraq were identified and contacted, via special platforms on social media specified for them, to answer a 30-item online survey.

**Results:**

We surveyed n = 255specialists (20% were women); interventional cardiologists constituted 44.7%, followed by cardiology trainees at 31%. Almost 30% reported having been involved in clinical trials, with data collection being the more frequently reported role (21.2%). Prior participation was not significantly associated with respondent gender, academic affiliation, or presence of institutional ethical committee. Of the total, 95.7% thought that clinical trials should be conducted in Iraq, with 58.8% reporting that they would participate if invited. The most common barriers to respondents’ participation in trials were lack of electronic health records (52.2% of those surveyed) and time (51.4%), followed by the requirement of additional follow-up visits or investigations (34.1%). The most common motivators were establishing electronic health records (86.27%), education and training of the general population about clinical trials (84.7%), and dedicated training for healthcare providers about clinical trial basics (84.3%).

**Conclusion:**

Our work helps pave the path to implementing a robust clinical trial ecosystem in Iraq. Institutional and financial factors and a lack of dedicated research time are related to the cardiovascular clinical trial lag in Iraq. Future qualitative research can help in getting a deeper understanding of what is needed to create the right infrastructure.

## Graphical Abstract

**Figure:**
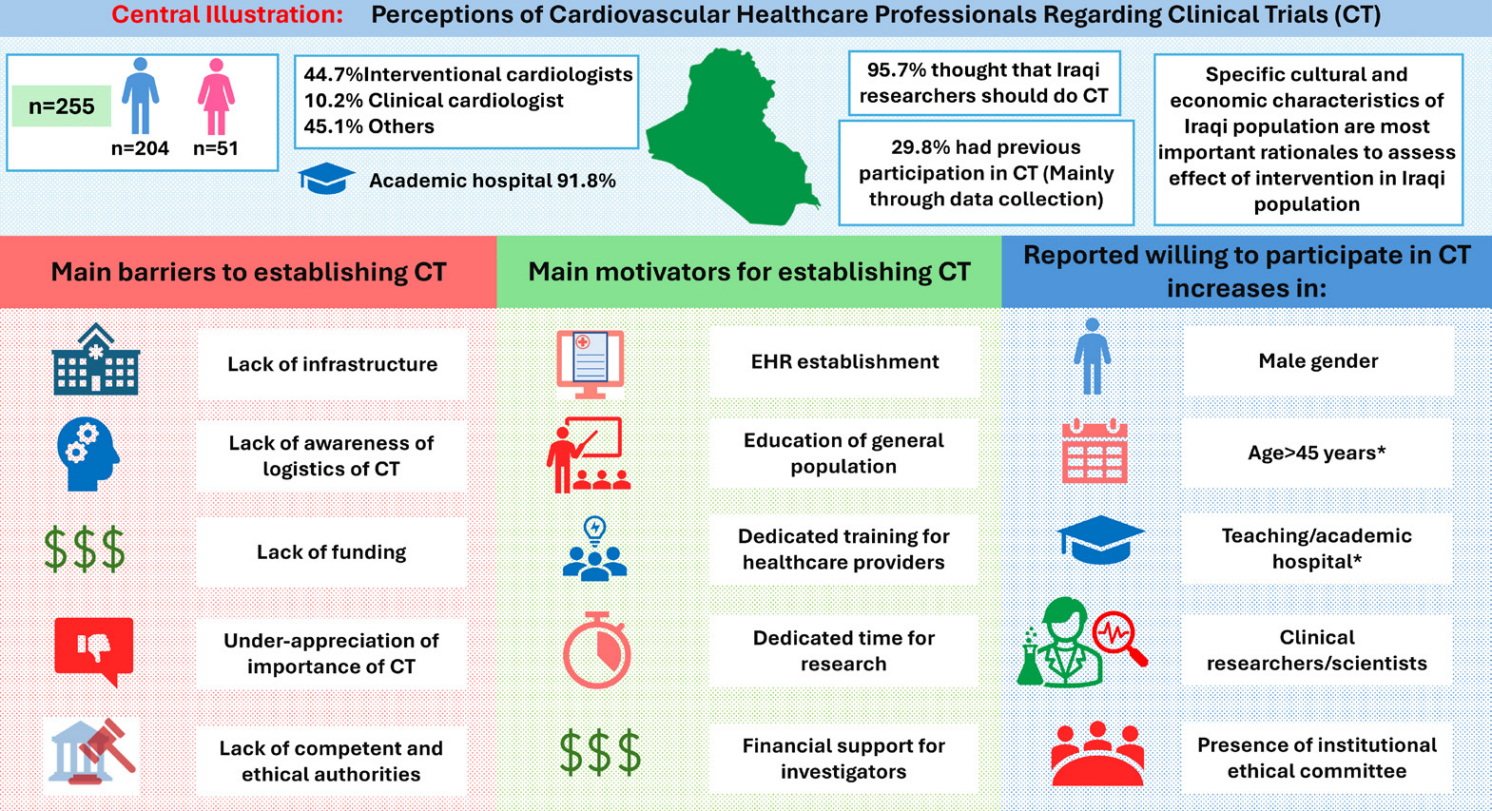
* Means that does not reach statistical significance

## Introduction

Low- and middle-income countries (LMICs), which account for 90% of the global disease burden, are significantly affected by cardiovascular diseases (CVDs) (1). Clinical trials are crucial for generating evidence-based strategies for managing CVDs, yet conducting such trials in LMICs is often hindered by financial, logistical, and regulatory challenges. (2). Even when conducted, there is dramatic geographical disparity, with most clinical trials being led and harbored by high income countries. Under-representation of LMICs in trials limits the generalizability of the results of existent trials. More importantly, LMICs need research that can form the basis for decisions to be made to improve their existent healthcare systems and to prioritize their limited resources (2). Iraq, like many other LMICs, has a poor research infrastructure and lack of research budget funding (3), not to mention absence of a regulatory framework for clinical trials. It is also unclear to what extent clinicians are trained to participate in clinical trials as investigators or co-investigators. All of these factors have resulted in the lack of an established clinical trial system in Iraq.

To establish a robust clinical trial ecosystem in such countries, it is essential to first gather data on the existing status of clinical trials, including the participation of healthcare professionals and the regulatory environment. Unfortunately, such data is scarce in the Middle East, and particularly in Iraq. Therefore, the primary aim of our study is to assess the perceptions of Iraqi cardiovascular healthcare professionals regarding clinical trials, identify potential barriers to their participation, and explore factors that may influence their involvement in future trials.

## Methods

### Target population

The survey was designed to be answered by cardiovascular HP, including physicians, allied healthcare professionals, or those in training involved in direct care of cardiac patients. The cardiovascular HPs were identified through specific social media groups for interventional cardiologists, clinical cardiologists, or healthcare professionals, such as Whats App and Viber groups. Furthermore, we identified all fellows in training in interventional cardiology and in cardiovascular imaging who were registered at the Iraqi Scientific Council of Cardiology, and they were contacted directly if were not listed in these social media groups.

### Survey development

The investigators developed a 30-item survey for cardiovascular HPs, in English, based on extensive literature review done by the study investigators who researched what insights, barriers, and motivators were reported in prior studies. Study investigators (ZAD, HAF, MP, and JL) did extensive revisions and designed the survey questions. The clarity and objectivity of the survey questions were checked by those investigators. Four healthcare professionals in Iraq who were potential respondents also answered the survey to check its clarity. All feedback was considered, and amendments were made to the survey, as required. ZAD then designed the final the Google Form, and a link to that form was shared with potential respondents.

The survey questions included (The list of items is reported in the Appendix in details):

1. Respondents’ characteristics, including age, gender, specialty, and affiliating institution.
2. Current and future insights of healthcare professionals regarding clinical trials in Iraq.
3. A 5-point Likert scale to assess the respondents’ perceptions regarding barriers for participation in clinical trials in Iraq.
4. A 5-point Likert scale to assess the respondents’ perceptions regarding motivators to initiate and/or participate in clinical trials in Iraq.

The survey invite was sent via social media (WhatsApp and Viber) to cardiovascular HPs in Iraq who could participate anonymously. They were contacted through official WhatsApp/Viber groups and/or personally to receive the survey link and responded voluntarily.

A paragraph at the start of the survey explained the aims of the study to the survey respondents and that completing the survey meant that they consented to participate. They were also reassured that the personal data would be kept anonymous and that responses will only be used for survey analysis which will be presented as scientific publication. The survey was carried out from September 2022 to mid-November 2022. The study is compliant with the national ethical protocols and ethical approval was obtained from Al-Kindy College of Medicine/University of Baghdad, with ethics number 194, dated at 22/06/2022.

## Statistical analysis

Descriptive statistics were used to analyze the survey responses, with frequencies expressed as percentages. Categorial variables were compared using the Chi-square test. IBM SPSS statistics version 26 was used for statistical analysis of respondents’ characteristics with their responses in univariate and multivariate regression analysis. Differences were considered significant if p < 0.05.

## Results

### Demographics

A total of 255 cardiovascular HPs responded, of which 114 (44.7%) were interventional cardiologists and 48 (18.82%) were allied healthcare professionals. No missing data was observed. The majority of respondents, 137 (53.7%), were aged 31–45 years. A fifth of respondents (n = 51) were women. Most of the respondents; n = 234 (91.8%); were affiliated with teaching or academic institutions. Of the total, 152 respondents (59.6%) reported that an active and functioning ethical committee authority is present at their institutions while 57 (22.4%) did not know if such authority was present at their institutions. Detailed demographics of respondents are shown in Table 1.

**Table 1:** Characteristics of healthcare providers that responded to the survey.


CHARACTERISTICS OF RESPONDENTS	NUMBER	PERCENTAGE

Age category (years)		

<30	45	17.60%

31–45	137	53.70%

46–55	54	21.20%

56–65	16	6.30%

>66	3	1.20%

Female sex	51	20%

Specialty		

Interventional cardiology	114	44.70%

Clinical cardiology	26	10.19%

Cardiac imaging/Echocardiography	25	9.80%

Electrophysiology	1	0.39%

Clinical researcher/Physician scientist	17	6.67%

Allied healthcare professional	48	18.82%

Cardiac-cardiothoracic surgery/vascular intervention	4	1.57%

Other medical specialties	19	7.45%

Medical student	1	0.39%

Designation/Role		

Cardiology consultant	74	29.01%

Non-cardiology consultant	17	6.67%

Cardiology fellow in training	79	30.98%

Non-cardiology fellow in training	19	7.45%

Allied healthcare professional	48	18.82%

Others	18	7.07%

Teaching/academic hospital	234	91.80%

Actively functioning ethical committee (authority) in the respondent facility		

Yes	152	59.60%

No	46	18%

The respondent did not know	57	22.40%


### Perceptions regarding if and why Iraqi researchers should conduct clinical trials in Iraq

**Figure 1:**
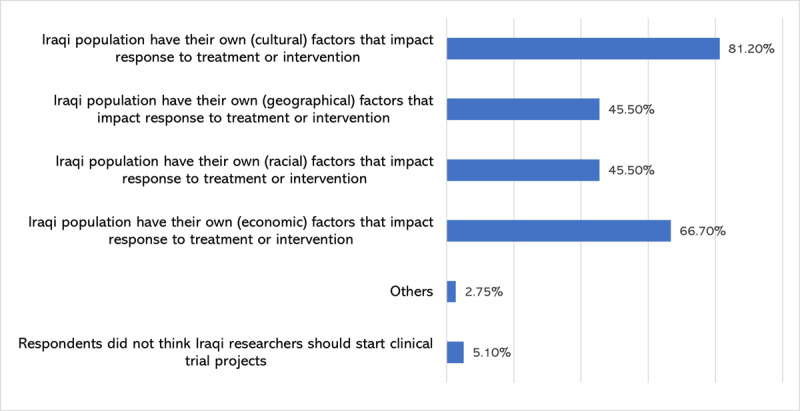
Insights of respondents toward conducting clinical trials in Iraq.

A total of 244 (95.7%) of the respondents thought that Iraqi researchers should conduct clinical trials in Iraq. The most frequently reported reasons for the need of clinical trials were cultural factors [reported by 207 (81.2%)] and economic factors [reported by 170 (66.7%)] may affect Iraqi population responses to treatment. Detailed distribution of these perceptions is shown in Figure 1.

### Perceived barriers to establishing clinical trials in Iraq

**Figure 2:**
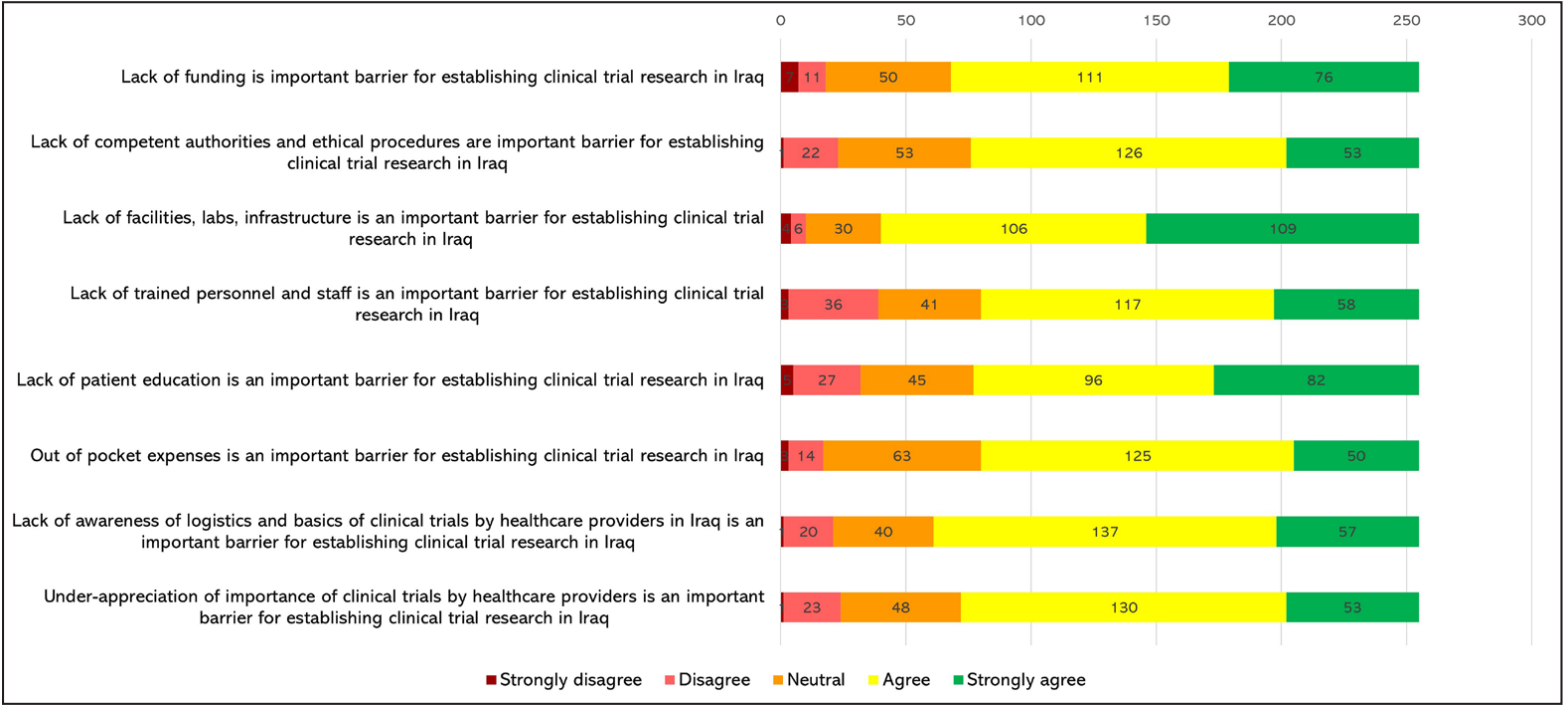
Perceived barriers to establishing clinical trials in Iraq.

Figure 2 reports the perceived barriers to setting clinical trials in Iraq. The most commonly agreed upon barrier was the lack of facilities, labs, and infrastructure; 215 (84.31%) agreed or strongly agreed. Furthermore, 187 (73.33%) agreed or strongly agreed that lack of funding is an important barrier and 194 (76.07%) agreed or strongly agreed that lack of awareness of logistics and basics of clinical trials by healthcare professionals was also an important barrier.

**Figure 3:**
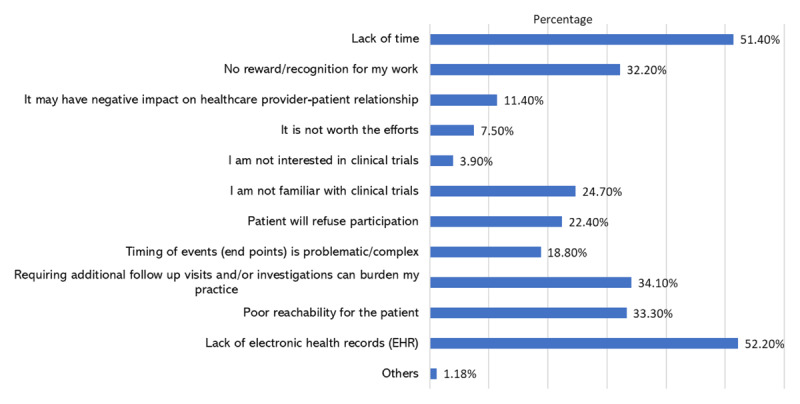
Barriers that might prevent cardiovascular healthcare professionals from participating in clinical trials if invited to participate as co-investigators.

The lack of electronic health records [52.2% (n = 133)] and time [51.4% (n = 131)] were reported as the most common barriers for participation in trials as a co-investigator (see Figure 3).

### Perceived motivators for establishing clinical trials in Iraq

**Figure 4:**
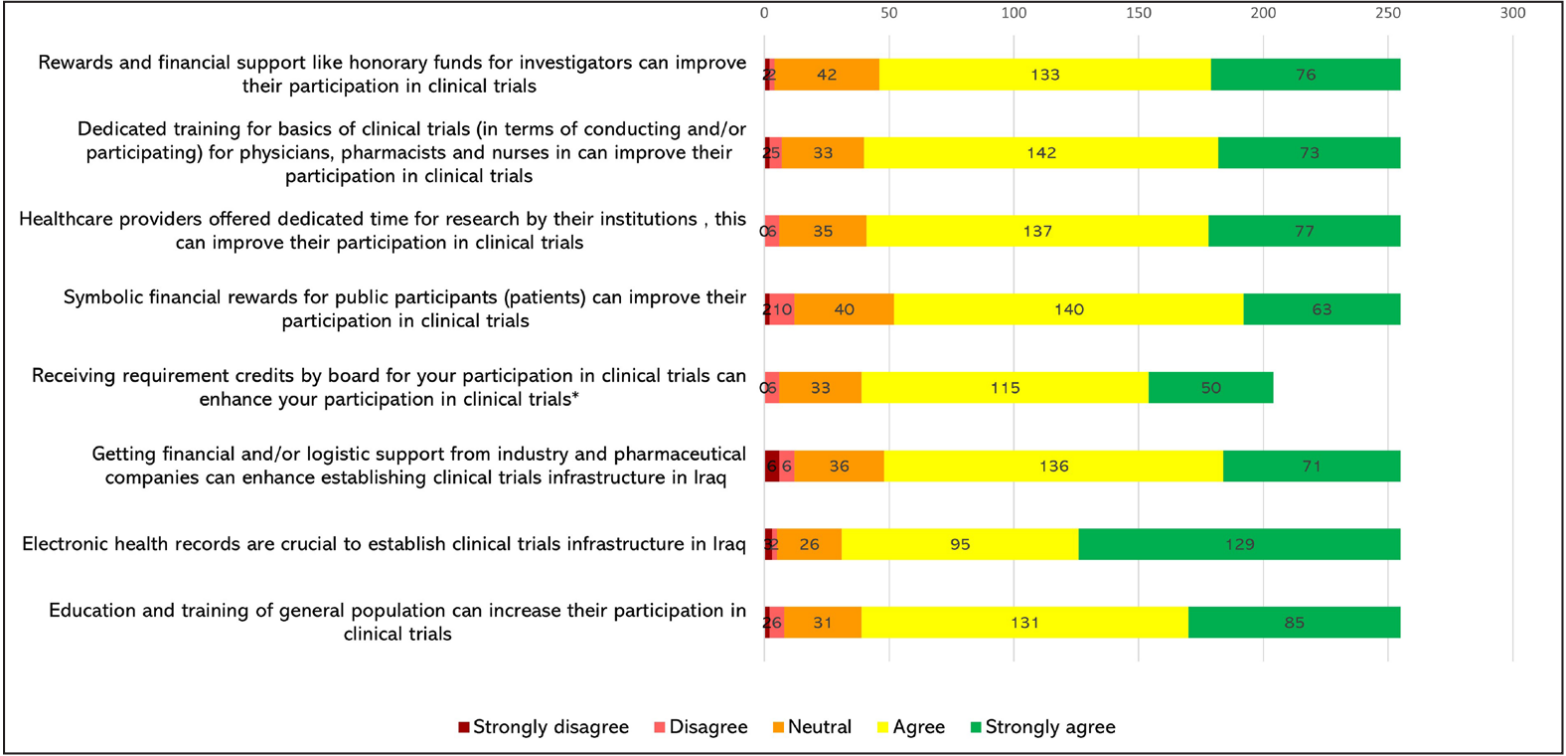
Perceived motivators for establishing clinical trials in Iraq. *We mean allocation of credits by Medical Board/Council for physicians or fellows that participate in clinical trials.

Figure 4 reports the perceived motivators to setting clinical trials in Iraq. Most of the respondents [87.84% (n = 224)] agreed or strongly agreed that electronic health records are crucial to establishing clinical trial infrastructure in Iraq. Of these respondents, 84.7% (n = 216) agreed or strongly agreed that education and training of the general population can increase population participation in clinical trials, while 84.31% (n = 215) of respondents agreed or strongly agreed that dedicated training for the basics of clinical trials for healthcare providers can improve their participation in trials.

### Previous and future participation of respondents in clinical trials

Table 2 reports the characteristics of respondents conditional on previous participation in trials. There was a significant difference in age distribution between those with previous participation but no other differences according to gender, type of hospital, presence of institutional ethical committee or specialty of respondents.

**Table 2:** Previous participation of respondents in clinical trials according to baseline characteristics.


FACTORS	YES	NO	

**Age**	**%**	**%**	

<30	15.6	84.4	0.033

31–45	29.2	70.8	

46–55	35.2	64.8	

56–65	56.3	43.8	

>66	33.3	66.7	

**Gender**			

Male	29.4	70.6	0.784

Female	31.4	68.6	

**Type of Hospital**			

Teaching/academic	29.9	70.1	0.897

non-teaching	28.6	71.4	

**Presence of institutional active ethical committee**			

Yes	31.6	68.4	0.42

No	32.6	67.4	

Respondent did not know	22.8	77.2	

**Specialty**			

Interventional cardiology	29.8	70.2	0.294

Clinical cardiology	30.8	69.2	

Cardiac imaging/Echocardiography	45.8	54.2	

Electrophysiology	100	0	

Clinical researcher/Physician scientist	41.2	58.8	

Allied health care professional	20.8	79.2	

Cardiac-cardiothoracic surgery/vascular intervention	25	75	

Other medical specialties	20	80	

Medical student	/	100	


**Figure 5:**
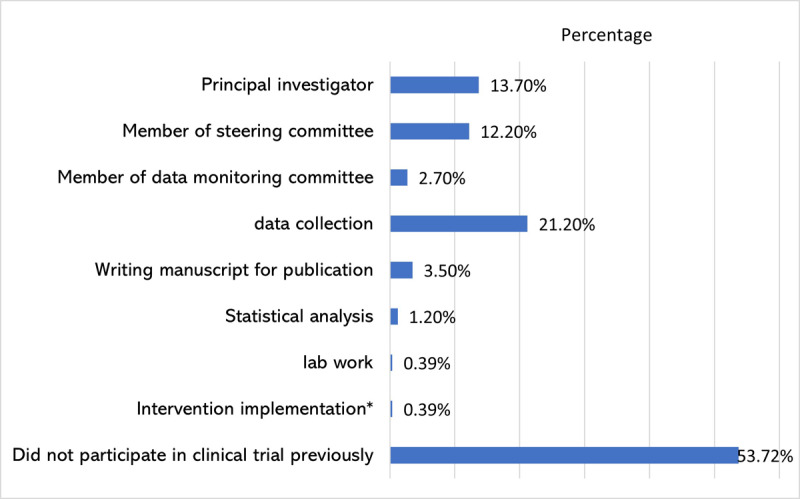
Previous role of respondents in clinical trials. Three respondents mentioned in the free text that their role in the clinical trial was published research, study-thesis, and head of department; these responses were not included in this figure. *One respondent mentioned in the free text that he was the device implanter of a clinical trial. **Some respondents chose (No) when they were asked if they had prior participation in clinical trials, yet they chose one of the roles when they were asked what your role in clinical trials was, we think that those respondents misinterpreted their participation in certain roles (mostly data collection) for research or interventional studies as participation in trials despite such studies are not necessarily were clinical trials.

Figure 5 shows the type of role of those who participated in previous trials. Hence, 29.8% (n = 76) reported prior participation in clinical trials with data collection being the most frequently reported role (21.2%).

Table 3 reports the respondents willing to participate in cardiovascular clinical trials in Iraq if invited; 150 (58.8%) respondents confirmed that they would participate as co-investigator if invited to participate in clinical trials, while 96 (37.6%) respondents reported that they cannot determine if they will or will not participate as co-investigators in future as this depends on many factors.

**Table 3:** Respondents willing to participate in cardiovascular clinical trials in Iraq if invited.


FACTORS	WILLING TO PARTICIPATE			

	YES	NO	CANNOT DETERMINE	p VALUE

**Age**	**%**	**%**	**%**	

<30	44.4	4.4	51.1	0.072

31–45	56.9	2.2	40.9	

46–55	72.2	7.4	20.4	

56–65	68.8	0	31.3	

>66	66.7	0	33.3	

**Gender**				

Male	61.3	4.4	34.3	0.043

Female	49	0	51	

**Type of Hospital**				

Teaching/academic	60.3	3.8	35.9	0.128

non-teaching	42.9	0	57.1	

**Presence of institutional active ethical committee**				

Yes	65.8	2	32.2	0.024

No	56.5	6.5	37	

Respondent did not know	42.1	5.3	52.6	

**Specialty**				

Interventional cardiology	62.3	3.5	34.2	0.012

Clinical cardiology	73.1	3.8	23.1	

Cardiac imaging/Echocardiography	33.3	4.2	62.5	

Electrophysiology	0	0	100	

Clinical researcher/ Physician scientist	88.2	0	11.8	

Allied health care professional	54.2	0	45.8	

Cardiac-cardiothoracic surgery/vascular intervention	75	0	25	

Other medical specialties	35	15	50	

Medical student	100	0	0	


**Figure 6:**
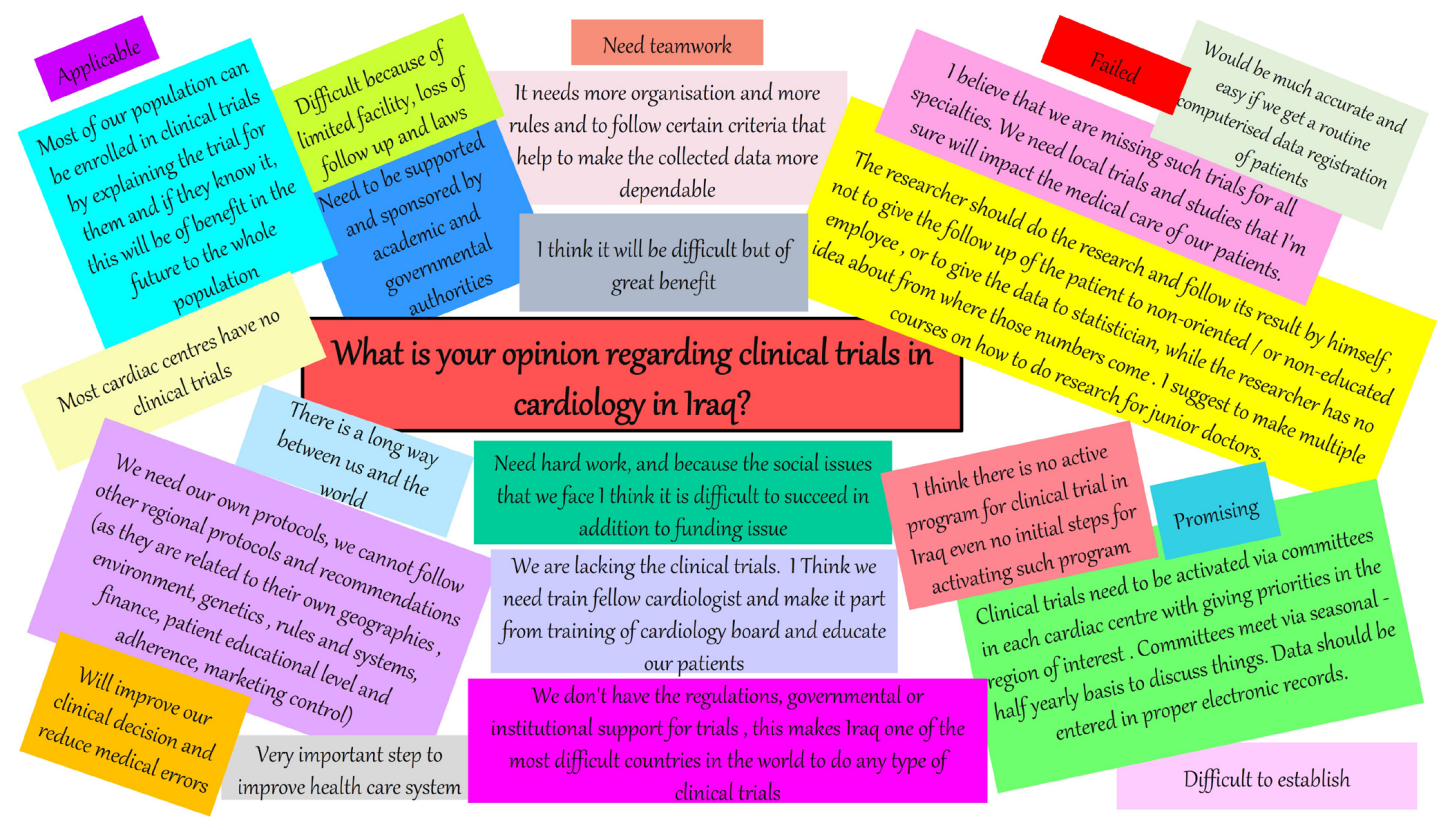
Qualitative data from the survey regarding cardiovascular healthcare providers opinions about clinical trials in cardiology in Iraq.

While gender, institutional-active ethical committees were significant factors for future participation in clinical trials in univariate analysis (Table 3), multiple regression analysis of variables predicting willingness to participate in future clinical trials in Iraq showed that presence of ethical committee in respondents’ institution was the only significant predictor for future participation, see Table 4. Further qualitative data from free text questions is expressed in Figure 6.

**Table 4:** Multiple regression analysis of predictors of respondents willing to participate in future clinical trials in Iraq*.


PREDICTOR	UNSTANDARDIZED COEFFICIENT B	STANDARD ERROR	95% CI LOWER BOUND	95% CI UPPER BOUND	p VALUE

Age	–0.124	0.078	–0.277	0.03	0.115

Gender	0.176	0.159	–0.137	0.488	0.269

Specialty	0.002	0.025	–0.048	0.052	0.951

Teaching/academic institution	0.0343	0.216	–0.081	0.768	0.112

Active institutional ethical committee	0.171	0.072	0.03	0.313	0.018

Prior participation in clinical trials	0.235	0.131	–0.022	0.492	0.073


*p = 0.002 for the regression model.

## Discussion

This cross-sectional survey is the first phase of the IRAQ-PPI Project (IRAQ-Patient Public Involvement Project) which encompasses three phases:

Phase 1: Assess the views of cardiovascular healthcare professionals regarding clinical trials in Iraq to understand how to pave the path to implementing a clinical trial ecosystem in Iraq (4).

Phase 2: Evaluate the knowledge of and assess the attitude and insights of Iraqi patients towards clinical trials in Iraq through survey data collected from a large cohort of Iraqi patients.

Phase 3: Establish the first PPI nucleus in Iraq through an intensive educational program to a cohort of general population through collaboration with the Iraqi Scientific Council of Cardiology to inform future cardiovascular research and trials.

After having the data that this survey added (phase 1) to the data that we will get from the survey that will be collected from patients during phase 2 (5), we will be able to portray and facilitate starting clinical trials in Iraq. This will become especially impactful when we start the third phase, which will be an educational program that will increase awareness of patients and population with the concept of clinical trials and PPI; this will support the clinical trial infrastructure in Iraq.

This project can provide the PPI toolkit that can be applied in other low-resource settings. From a professional aspect, this project is innovative not only as a cornerstone to establish a clinical trial ecosystem from scratch in Iraq but also in harnessing the emergence of medical students (who received proper education and teaching for this project) as an active research workforce in Iraq, which is unprecedented before this project. Such inclusion will enhance their career and will form a vital Iraqi nucleus in cardiovascular research if this process of recruiting young passionate talents continues. So far, IRAQ-PPI includes six medical students and two junior doctors distributed in different phases of this project.

To the best of our knowledge this is the first study from the Middle East regarding insights of healthcare professionals toward participation in clinical trials in the cardiovascular field. Furthermore, it is the first study from Iraq to gain an overview of the perceptions of healthcare professionals regarding clinical trials in cardiovascular disease. Understanding health professionals’ views is crucial as the healthcare infrastructure in Iraq presents significant challenges to develop a robust clinical trial system. Our study provides important key factors that can impact the contribution of cardiovascular healthcare professionals to clinical trials in Iraq, while suggesting crucial steps to promote their future participation.

One of the noteworthy takeaways of our study is the overwhelming support for clinical studies to be run in Iraq. Our results are similar from studies conducted by Al-Tannir et al., Sumi et al., and Ihara et al. in Saudi Arabia and Japan (6,7,8), where most physicians (88.4%, 96.1%, and 90%, respectively) expressed interest in conducting clinical trials. This supportive attitude among physicians might underscore a culture of cooperation within the medical community in those countries where clinicians recognize the importance of advancing medical knowledge collectively through these research endeavors. However, the favor toward the necessity of establishing clinical trials ecosystem among our survey respondents was mostly attributed to the existent cultural and economic factors. For example, Iraqi researchers would have a greater sensitivity to the distinctive cultural background that might impact patient behavior and adherence to therapy. Moreover, they might be more apt to consider the financial factors that impact the viability of the experiment and the willingness of patients to participate.

Despite the willingness expressed by HPs to conduct clinical trials; several issues were identified. Overall, the majority see each of the barriers listed as a significant problem. Although some of the barriers have more endorsement, this shows that it is multifactorial and gives an indication of how complex it is to address. The lack of adequate facilities, infrastructure, and funding emerged as significant obstacles. The hospitals in Iraq exhibit inconsistent quality, thus, recruiting patients from Iraqi hospitals for clinical trials may prove challenging primarily due to the suboptimal quality and limited infrastructure of these medical facilities. The disparity in quality across hospitals, coupled with a scarcity of facilities can complicate the recruitment process. In addition, several other challenges included ethical and regulatory system difficulties, inadequate research environment, operational constraints, and lack of patient education in clinical trials. Thus, raising the difficulty in obtaining participant patients in these trials. Our findings are consistent with challenges faced in low-resource settings and developing countries globally, where limited resources hinder research activities (1,9). However, this may differ partially when it comes to the barriers faced by developed countries like Japan and Germany, where most challenges included lack of time, simultaneous involvement with patient care, the required paperwork, and the need to prepare and manage the study documents (1,10,11). In an analysis of 156 studies assessing the barriers to participating in clinical trials in Europe, inadequate knowledge of trial methodology, lack of funding, and excessive monitoring were the main barriers. However, restrictive privacy laws and complex regulatory and ethical requirements also played critical roles in hindering participation in clinical trials in Europe (12).

In addition, another important barrier that our participants have highlighted is healthcare practitioners’ limited knowledge of the operational and fundamental aspects of clinical trials. This highlights the significance of educational efforts to improve research literacy among HPs. These efforts might include implementing training programs and seminars focused on clinical trial techniques and ethical issues, so healthcare practitioners can acquire the necessary information and skills to actively engage in research activities, thus closing the existing knowledge gap and enabling their participation in research activities. Prior experiences in initiating programs of research training, networking, and mentorship enabled developing countries in having a platform for sustainable clinical trials and research centers (13).

On the other hand, we also examined the factors that would motivate HPs to engage in clinical trials. Interestingly, electronic health records were found to be the most important aspect of developing a clinical trial infrastructure. In our opinion, this issue is of utmost significance, yet it is frequently overlooked in terms of patient management and organization of their information and records inside the Iraqi healthcare system. Currently, most Iraqi healthcare institutions and hospitals utilize a paper-based approach to document patients’ data (14,15), with negligible utilization of electronic records. Insufficient data collection and processing hinder the ability to derive significant findings and observations from real-world data. Globally, electronic health records have become a must-be present element in any healthcare infrastructure. With the emerging role and preference of pragmatic and electronic health records-based trials (16), it becomes clear that electronic health records should be a priority for healthcare decision-makers and stakeholders to establish a robust clinical trial ecosystem in Iraq especially if we want the process of data collection, outcome adjudication, and variables extraction to become streamlined and pragmatic.

While nearly 30% of respondents reported prior participation in clinical trials, a significant portion remained undecided about future participation. This percentage is not only low compared to previous studies (6,7,8,11), but also might not be accurate, as the investigators of this study did a thorough literature review and can hardly find published clinical trials from Iraq in the cardiovascular field. We theorize that either the respondents were part of international trials (which were very scarce), the respondents were misinterpreting the term (clinical trials) as other forms of interventional studies, or these trials have never been published. In the study by Sumi et al. in Japan (7), among respondents, 68% of physicians reported current participation in clinical research; 74% reported past participation in clinical research. Physicians, due to their profession, tend to have a greater awareness of, involvement in, and support for clinical research initiatives. This increase in participation rates may be attributed to various factors, such as professional obligation, a greater awareness of the significance of clinical research, and improved availability of information on ongoing studies.

Differences in participation also existed depending on demographic and professional attributes. Younger participants and individuals in specialized fields like clinical cardiology and clinical research were more likely to engage in clinical trials. This is different to what Sumi et al. reported (7), where there was a greater number of faculty members, as compared to residents or doctorate students, who stated their previous, current, and future involvement in clinical research. Women respondents showed less willing to participate in future trials; this issue needs to be addressed and overcome properly by raising awareness about the value of women in clinical research, ensuring women have equal access for training and leadership roles in clinical trials. This can be achieved by mentorship opportunities. Institutional policies should ensure supportive work environments to empower women to pursue research career.

In our study, the main predictor for future participation in clinical trials was the existence of an ethics committee at the respondent’s institution. This emphasizes the crucial importance of ethical oversight in promoting research involvement. Ethics committees are vital in ensuring that research is conducted responsibly and ethically (17). They are responsible for safeguarding the rights and well-being of participants and maintaining the integrity of the research process. Their presence not only establishes a structure for ethical governance but also serves as a clear indication to researchers and potential participants that the institution places a high importance on ethical standards (17,18). This can enhance trust and encourage greater willingness to participate in research projects.

Future research is needed regarding how targeting specific barriers can improve the clinical trials ecosystem in Iraq like establishing electronic health records in usual healthcare settings.

## Limitations

While our study provides valuable insights into the perceptions of HPs regarding clinical trials in Iraq, we should acknowledge several limitations. Firstly, our survey was conducted using social media platforms, which may increase the risk for selection bias as it could exclude HPs who do not use these platforms or who are less active online. Secondly, our survey relied on self-reporting by respondents. This may encourage the participants to provide socially likeable and desirable responses or to overestimate their willingness to participate in clinical trials. Thirdly, even though cardiovascular specialists in Iraq are still of limited number and this survey included most of them, the numbers are still small, which might affect the reliability of the statistical analysis. Lastly, the survey was conducted in English, which may have posed a language barrier for some potential participants.

## Conclusion and future directions

**Figure 7:**
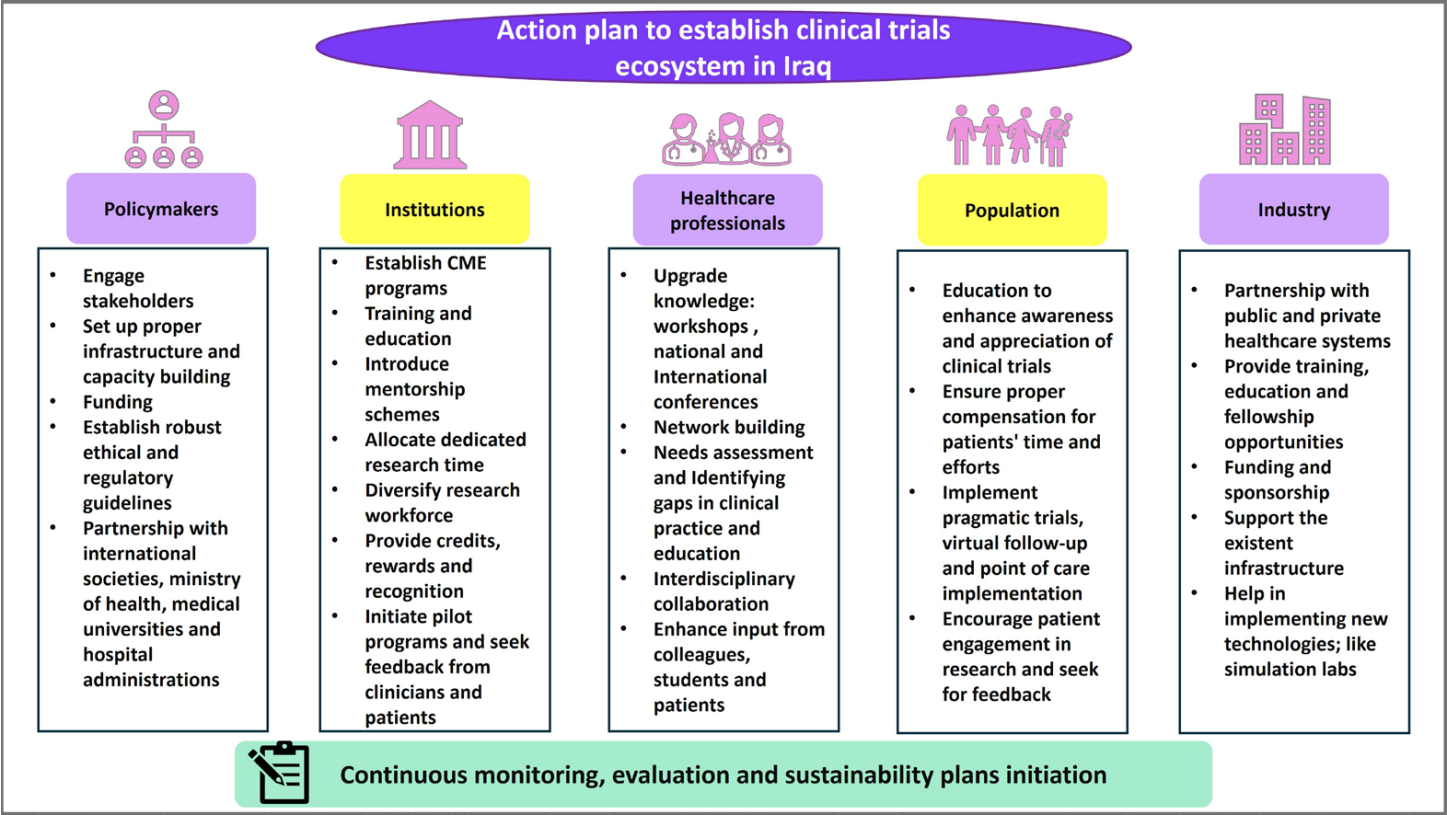
Action plan to establish clinical trials ecosystem in Iraq.

Despite the acknowledged importance of conducting clinical trials within Iraq, our findings reveal significant barriers that hinder physicians from participating in such trials. Institutional and financial constraints, coupled with a lack of dedicated research time and infrastructure with very limited facilities, pose formidable challenges to the advancements of conducting these trials in Iraq. It is crucial for the Iraqi government and health authorities to develop and implement a comprehensive regulatory framework for clinical trials. This should include guidelines for trial approval, ethics review processes, and safety monitoring to ensure that clinical trials meet international standards. In addition, we recommend that healthcare institutions invest in training programs to improve the research and clinical trial skills of healthcare professionals, particularly in the cardiovascular field. This would increase the capacity of local researchers to lead and participate in trials. Finally, to increase participation in clinical trials, awareness campaigns should be launched targeting both healthcare professionals and the public. These campaigns can highlight the importance of clinical trials for improving healthcare outcomes and demonstrate the potential benefits for patients and researchers alike. Addressing these barriers will require concerted efforts from stakeholders including policymakers, healthcare institutions, and research organizations. Investments in healthcare infrastructure, capacity building, and research literacy programs are essential to further improve a and foster a conductive environment for clinical trial conduct in Iraq. Additionally, initiatives to raise awareness among healthcare professionals about the importance and logistics and basics of clinical trials are crucial steps toward overcoming such challenges; see Figure 7 summarizing the proposed action plan to establish a clinical trials ecosystem in Iraq.
